# Research progress in the treatment of lipid metabolism disorder in patients with diabetic kidney disease by the integrated traditional Chinese and Western medicine

**DOI:** 10.3389/fendo.2025.1631312

**Published:** 2025-07-30

**Authors:** Lingli Sheng, Ziyi Cao, Lin Wang, Youhua Xu, Dingkun Gui

**Affiliations:** ^1^ Faculty of Chinese Medicine, Macau University of Science and Technology, Macao, Macao SAR, China; ^2^ Nephropathy Department II, Longhua Hospital Shanghai University of Traditional Chinese Medicine, Shanghai, China; ^3^ College of Integrated Chinese and Western Medicine, Shaanxi University of Chinese Medicine, Xianyang, China; ^4^ Department of Nephrology, Shanghai Sixth People’s Hospital Affiliated to Shanghai Jiao Tong University School of Medicine, Shanghai, China

**Keywords:** diabetic kidney disease, lipid metabolism disorder, traditional Chinese patent medicines, Chinese medicine compound, single Chinese medicine, integrated traditional Chinese and Western medicine

## Abstract

Diabetic kidney disease (DKD) is one of the most common and severe chronic microvascular complications of diabetes mellitus (DM). The pathogenesis of DKD is complex, and lipid metabolism disorders play an important role in the pathogenesis of DKD. DKD belongs to the category of “kidney deficiency”, “edema”, “guan ge” and other pathological factors secondary to “thirst quenching disease” in traditional Chinese medicine. The pathological factors mainly focus on blood stasis and toxicity, which is consistent with modern medical theory. At present, the efficacy and safety of integrated traditional Chinese and Western medicine in treating lipid metabolism disorders in patients with DKD have been extensively studied and confirmed. In this review, the application and possible mechanism of traditional Chinese patent medicines (Bailing Capsule, Shenyan Kangfu Tablets, Jinshuibao Capsule, Huangkui Capsule, Yi-Shen-Hua-Shi granule, Shenmai injection), Chinese medicine compound (Tangshen Formula, Danggui Buxue Decoction, Tangshenkang), single Chinese medicine (Astragalus membranaceus, Panax notoginseng, Salvia miltiorrhiza) combined with Western medicine in the treatment of DKD with lipid metabolism disorder were discussed, in order to provide ideas for clinical diagnosis and treatment of patients with DKD with lipid metabolism disorder.

## 1 Introduction

Diabetic kidney disease (DKD) is a serious microvascular complication of diabetes mellitus (DM) (1). With the increase of global prevalence of diabetes, about 30%~40% of DM patients will progress to DKD, which is the most common cause of end-stage renal disease (ESRD) (2). Therefore, early screening, control, and treatment of DKD is extremely important. The treatment strategy for early DKD is not just about controlling blood glucose, other related pathogenic factors cannot be ignored. The Chinese Guideline for the Prevention and Treatment of Type 2 diabetes (2020) proposed that lipid metabolism disorder is one of the risk factors of DKD, which should be paid more attention to and controlled.

Lipids are the significant component of the cell membrane, playing a crucial part in energy generation, cellular homeostasis, cell signal transduction and survival (3). Lipids have duality. Moderate increase has a positive effect, while overdose can arise oxidative damage, resulting in tissue lipid peroxidation and ultimately lipid toxicity. Lipid homeostasis is prominent in preventing lipid toxicity (4). Modern pathology believes that lipid deposition in the kidneys has become a momentous factor leading to renal damage in DKD. High levels of lipids in the human body can provoke renal tissue fat deposition, glomerulosclerosis, and mesangial dilation, exacerbating proteinuria and progression of tubulointerstitial fibrosis, and directly damaging podocytes, leading to irreversible damage to renal function (5, 6). Thus, regulation of lipid metabolism disorder is vital significance for reducing the incidence rate of lipid metabolism disorder in DKD and slowing its progress.

In recent years, molecular biology has been developing rapidly, With the widespread application of ultra-high performance liquid chromatography and mass spectrometry, the active ingredients, indication and mechanism of traditional Chinese medicine (TCM) have been further studied in depth. As primary or alternative therapy for DKD, TCM has shown good clinical efficacy. More and more studies emphasize the identification of bioactive compounds in TCM and the molecular mechanisms of renal protection (1).

This review aims to discuss the integrated treatment of traditional Chinese and Western medicine for lipid metabolism disorders in patients with DKD, emphasize the significance of prevention and treatment of DKD and the importance of simultaneous regulation of lipid metabolism disorder in the treatment process; TCM will play a leading part in the treatment of such diseases with its advantages of multi-target, multi-pathway, and compound efficacy. Then, we hope this review could provide reference for clinical diagnosis and scientific research.

## 2 The pathogenesis of DKD combined with lipid metabolism disorders

DKD patients typically suffer from lipid metabolism disorders, and dyslipidemia occurs throughout the entire process of illness (7). In the early stage of DKD, hypertriglyceridemia develops. Then overt DKD patients with macroalbuminuria have elevated levels of total cholesterol (TC) and significantly increased low-density lipoprotein (LDL) cholesterol. And renal failure increases lipoproteins rich in triglyceride (TG) (such as chylomicrons and very low density lipoprotein) and decreases high-density lipoprotein (HDL) cholesterol (8, 9). Ectopic lipid deposition refers to the excessive accumulation of lipids, notably TGs, in lean tissues like the kidneys, causing lipotoxic damage and then gives rise to a series of pathological and physiological changes (10).

The kidney is rich in mitochondria so that it is a highly active metabolic organ with high energy demands (11). The demands for ATP vary in different regions of the kidney, with glucose being the preferred energy matrix for renal substrates. The glomerulus tends to use glucose, while the tubules prefer fatty acids (FAs) (12). Podocytes, also known as glomerular epithelial cells, form a silt diaphragm (SD) with their foot processes, which are a portion of the glomerular filtration barrier (GFB) and restrict the filtration of albumin into urinary space (13). SD is assembled in lipid rafts, which are special membrane domains rich in cholesterol and sphingolipids (14, 15). However, lipid accumulation can induce podocyte hypertrophy, the disappearance of podocyte processes and apoptosis, causing irreversible damage to podocytes, resulting in a large amount of protein entering the urine and intensifying the progression of DKD (16). Renal tubular cells and podocytes are prone to lipid accumulation, inducing mitochondrial stress, inflammation, insulin resistance (IR), endoplasmic reticulum stress (ERS), and ultimately causing cell death (17). Under physiological conditions, TGs from the diet are decomposed into free fatty acids (FFAs) and glycerol in the cytoplasm. Fatty acid transporters are mainly responsible for FFA transport in renal tubular cells, for instance, fatty acid transport protein-2(FATP2) (18). And B-class scavenger receptors, such as CD36, mainly focus on FFA transport in podocytes (19). After being stimulated by palmitate (a saturated FFA), the expression of CD36 is upregulated in podocytes, speeding up the transport of CD36 from the cytoplasm to the cell membrane, then enhancing the lipid uptake of podocytes, leading to oxidative stress (20). Fatty acids (FAs) undergo beta oxidation in cells, also called FA oxidation (FAO), then form acetyl-CoA or are stored in the form of lipid droplets (LD), while glycerol directly enters the glycolytic pathway (21). Both incomplete (FAO and lipid peroxidation can bring about oxidative stress, endoplasmic reticulum stress, and activation of pro-inflammatory processes (22). If too much acetyl-CoA is produced, the tricarboxylic acid cycle (TCA cycle) will overload, provoking the conversion of acetyl-CoA to ketone bodies, which can be taken for a fuel origin at low glucose levels. Nevertheless, if glucose levels are high, excess acetyl-CoA can be transformed into FAs, TG, cholesterol, steroids, and bile salts, a process known as adipogenesis (21). That is, long-term hyperglycemia in diabetes patients will aggravate fatty acid synthesis and TG accumulation.

**Figure 1:**
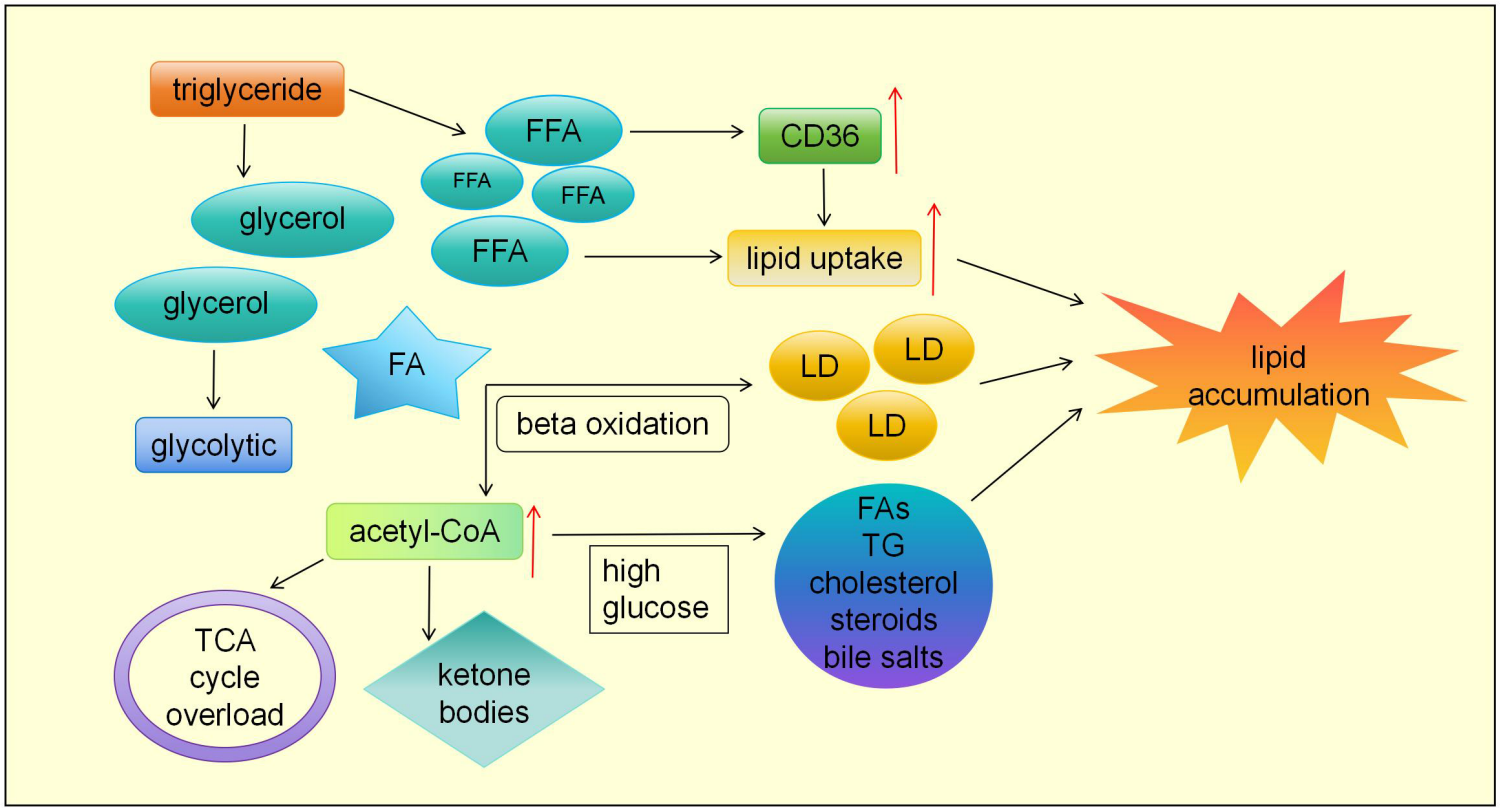
The mechiasm of lipid accumulation. TGs from the diet are decomposed into free fatty acids (FFAs) and glycerol in the cytoplasm. After being stimulated by FFA, the expression of CD36 is upregulated in podocytes, speeding up the transport of CD36 from the cytoplasm to the cell membrane. Fatty acids (FAs) undergo beta oxidation in cells, also called FA oxidation (FAO), then form acetyl-CoA or are stored in the form of lipid droplets (LD), while glycerol directly enters the glycolytic pathway. If too much acetyl-CoA is produced, the tricarboxylic acid cycle (TCA cycle) will overload, provoking the conversion of acetyl-CoA to ketone bodies. if glucose levels are high, excess acetyl-CoA can be transformed into FAs, TG, cholesterol, steroids, and bile salts, a process known as adipogenesis.

What’s more, sterol regulatory element binding proteins (SREBPs) are transcription factors participating in regulating lipid biosynthesis, among which SREBP-1a and SREBP-1c preferentially activate genes involved in fat production, while SREBP-2 is mainly responsible for transcriptional regulation of cholesterol homeostasis related genes (23). In the kidney, the increase of TG level is related to the increase of SREBP-1c expression level (24). Peroxisome proliferator activated receptor (PPAR)α, PPARβ/δ, and PPARγ are members of the ligand activated transcription factor nuclear receptor family (25). PPARα, a ligand dependent nuclear receptor, takes part in the process of lipid metabolism, which is sensitive to exogenous compounds such as FAs, and regulates FAO like PPARβ/δ (26, 27). PPARγ has two contrary effects on lipid metabolism. On the one hand, the elevation of PPARγ can bring about the expression of lipoprotein lipase (LPL), causing fat breakdown, reducing TG and LDL-C levels, then improving lipid imbalance (28). On the other hand, PPARγ activation can promote CD36 expression so that lipid deposition occurs in renal tubular cells (29). But the specific mechanism needs additional experimental verification. Furthermore, peroxisome proliferator activated receptor gamma coactivator-1α (PGC-1α) is related to mitochondrial biogenesis and fatty acid beta oxidation directly. Inhibiting PGC-1α can significantly accelerate the reduction of FAO and increase lipid accumulation (**Figure 1**) (30, 31). Meanwhile, PGC-1α can strengthen the expression of farnesoid x receptor (FXR) to regulate triglyceride metabolism (32). Accordingly, overexpression of PGC-1α can protect renal cells from lipotoxicity to a certain extent and play an important role in the pathogenesis of DKD. Moreover, stearoyl-CoA desaturase (SCD) is a rate limiting enzyme that converts saturated FAs into monounsaturated FAs, resulting in the formation of neutral LD (33). Overexpression of SCD1 can reduce apoptosis of proximal renal tubular epithelial cells induced by saturated FA (34).

HDL is a cholesterol reverse transporter protein which can unrestricted enter and exit the arterial wall, uptake harmful substances such as LDL, cholesterol, TGs, etc. deposited in the inner layer of the vascular wall, and transport them to the liver for decomposition and excretion (35). Apolipoprotein (APO) A-I is the main component of HDL. Studies have found that it can increase glucose tolerance and insulin sensitivity, inhibit liver gluconeogenesis, reduce liver glycogen output, and have an important protective effect on diabetes and its complications (36). At the same time, the decrease in HDL levels downregulated the levels of circulating APOM, APOE and APOL1 (21). ATP-binding cassette transporter A1 (ABCA1) controls cholesterol efflux by transporting free cholesterol and phospholipids from the cell to high-density lipoprotein particles containing APOA-I (37). The expression of ABCA1, participated in impaired cholesterol efflux, declines in DKD patients’ podocytes (38). Studies have shown that the deletion of Subtilisin/kexin type 9 serine protease (PCSK9) can improve dyslipidemia (39). Especially in the high-fat diet animal model of kidney injury, the reduction of PCSK9 level can promote renal lipid accumulation caused by CD36 (40). PCSK9 can reduce the level of LDL receptor on plasma membrane, thereby promoting cholesterol influx of circulating LDL (21). Elevated plasma LDL levels and the formation of foam cells (macrophages that ingest LDL) stimulate the release of pro-inflammatory cytokines and accelerate the inflammatory response, thereby causing kidney injury by affecting lipid metabolism and causing oxidative stress (41). LDL size is negatively regulated by serum TG levels, and LDL size is significantly reduced in hypertriglyceridemic subjects (42). Ox-LDL is easier to deposit on the inner wall of blood vessels than normal LDL, leading to atherosclerosis and other cardiovascular complications. Klotho is an important renoprotective protein, which can effectively eliminate renal ox-LDL deposition through IGF-1R/RAC1/OLR1 signaling axis, thereby improving podocyte injury (43). Liver x receptors (LXRs) are ligand activated nuclear receptor transcription factors that play a vital part in regulating cholesterol and triglyceride metabolism by regulating the expression of fatty acid synthesis related genes such as SREBP1-c (24). In addition, the LXR pathway may regulate the expression of ABCA1 gene through PPAR (28). FXR is a multifunctional transcription factor that regulates bile acid homeostasis and glucose and lipid metabolism in various tissues (44). It can also alleviate renal fibrosis (45), regulate mitochondrial biogenesis pathways (46), and reduce TGFβ-SMAD signal transduction and inflammatory response in renal mesangial cells (47), deferring the progression of DKD (48).

## 3 Integrated traditional Chinese and Western medicine treatment for DKD combined with lipid metabolism disorder

As mentioned earlier, DKD combined with lipid metabolism disorders involves multiple mechanisms such as lipid deposition, hyperglycemia, oxidative stress, inflammation, and fibrosis. At present, Western medicine clinical practice mainly reduces kidney damage and delays the progression of kidney disease by controlling blood lipids, blood sugar, and blood pressure levels.

At present, the clinical therapeutic drugs in Western medicine mainly include the following categories: fibrates, HMG-CoA reductase inhibitors (statins), proprotein convertase subtilisin-kexin type 9 (PCSK9) inhibitors, sodium-glucose transporter-2 inhibitors (SGLT2i), glucagon-like peptide-1 (GLP-1) receptor agonists, renin angiotensin aldosterone system (RAAS) blockers etc (49).

Firstly, as an activator of PPARα, fibrates can improve fatty acid β oxidation and lipid accumulation in DKD mice through AMPK/FOXA2/MACD pathway, and alleviate apoptosis of renal tubular cells (50). However, clinical trials have shown that fibrates have an improving effect on proteinuria, but further research is needed to investigate the impact of fibrates on eGFR (49). Secondly, it has been confirmed that statins reduce the risk of cardiovascular disease in patients with DM and hypertension (51). Studies have shown that statins can improve proteinuria and reduce estimated glomerular filtration rate (eGFR) decline rate in DKD patients (52, 53). However, the specific impact on DKD is still unclear. Then, PCSK9 inhibitors are considered as alternative or adjuvant drugs to statins, but there is insufficient data on the impact of PCSK9 inhibitors on the progression of DKD (49). In addition, the strong protective effect of anti-diabetic drug, SGLT2i, on the kidney has been proved, and its mechanism may include anti lipid toxicity (54). GLP-1 receptor agonists can reduce blood lipids and alleviate renal ectopic lipid deposition in DKD rats (55). At the same time, it can also improve the decrease in urinary albumin and significantly slow down the decrease in eGFR in type 2 DM patients (56). RAAS blockers have been used as therapeutic drugs for DKD for decades (57). Commonly used drugs include angiotensin converting enzyme inhibitors (ACEI), angiotensin receptor blockers (ARB), and mineralocorticoid receptor antagonists (MRA), all of which can improve proteinuria and kidney injury. However, it should be noted that spironolactone and eperenone have a risk of hyperkalemia (58).

According to the theory of TCM, lipid metabolism disorder belongs to the categories of “phlegm turbidity”, “qi stagnation” and “blood stasis”. These pathological factors can lead to impaired blood flow and poor circulation of qi and blood. Over time, there is turbidity and blood stasis in blood vessels, obstruction of the venation, which can then lead to various diseases. The pathogenesis of DKD is consistent with it, that is, phlegm turbidity affects the normal consolidation and drainage of the kidney, causes proteinuria, hinders the blood flow of the kidney for a long time, forms blood stasis, aggravates the burden of the kidney, and accelerates the disease process (59).

### 3.1 Traditional Chinese patent medicines combined with Western medicine in the treatment of DKD with lipid metabolism disorder

Traditional Chinese patent medicines refer to a kind of medicine with certain specifications which can be directly taken under the guidance of the theory of TCM, with Chinese herbal medicine as the raw material, according to the prescribed prescription compatibility and preparation standards.

#### 3.1.1 Bailing capsule

Bailing Capsule is a capsule made from fermented Cordyceps sinensis powder (C. sinensis), mainly composed of mannitol, adenosine, and total amino acids. Its main functions are to nourish the lungs and kidneys, benefit essence and qi, and be commonly used as an adjuvant therapy for chronic renal insufficiency in clinical practice (60). It has anti-inflammatory, antioxidant, and proteinuria reducing functions (61). In addition, Bailing capsules are also used to treat chronic bronchitis, improve symptoms such as cough, asthma, and lower back pain in patients (60, 62). Multiple clinical studies have demonstrated that BC can deter the inflammatory process by reducing the expression of inflammatory factors such as hypersensitive C-reactive protein (hs-CRP), tumor necrosis factor-α (TNF-α) and interleukin(IL)-18, improve oxidative stress function, like a significant increase in superoxide dismutase (SOD) levels and a significant decrease in malondialdehyde (MDA) levels, reduce urinary protein, improve lipid metabolism and protect renal function (63, 64). Wang Wenru et al. (65) found that BC is superior in reducing TG through meta-analysis. A molecular biology study has shown that BC inhibits adipogenesis through triggering SCD and suppressing fatty acid synthase (FASN) expression, activates PPARα pathway and downstream acyl-coenzyme A oxidase 1 (ACOX1), a lipolytic enzyme regulated by PPARα in lipid oxidation, to enhance lipolysis, thereby delaying on the progression of DKD in rats and protecting renal function (66).

ARB are used to treat different types of hypertension, such as irbesartan, losartan, valsartan, etc. This class of drugs inhibits the production of angiotensin II, dilates the renal arteries, improves the state of hyperfiltration, hyperperfusion, and high pressure in the glomeruli, and significantly reduces the excretion of urinary protein (67). When BC is used in combination with ARB drugs, it can effectively improve the renal function of patients with DKD, reduce urinary protein excretion, and improve lipid metabolism disorders (68, 69). Dapagliflozin is a sodium-glucose cotransporter 2 inhibitor (SGLT2i), and the incidence of adverse reactions in the treatment of chronic kidney disease is relatively low. The combination of BC and dapagliflozin in the treatment of DKD can enhance the therapeutic effect, reduce renal injury, and delay the progression of the disease (70). Pancreatic kininogenase can prevent platelet formation and thrombosis, balance the hemodynamic disorders caused by the activation of the RAAS, reduce the leakage of urinary protein, and delay the progression of DKD. The combination of BC and pancreatic kininogenase can effectively improve hypertriglyceridemia in patients with early-onset DKD, reduce the level of proteinuria in stage III of DKD, and there are no adverse reactions during the medication period (71). Simvastatin and atorvastatin are commonly used statin drugs in clinical practice. When each of them is used in combination with BC, the improvement of blood lipids in DKD patients is better than when used alone, and it can significantly improve renal function and reduce the inflammatory response (72, 73). ,

#### 3.1.2 Shenyan Kangfu tablets

SKT is composed of thirteen traditional Chinese medicines, including Salvia miltiorrhiza Bunge, Panax ginseng, Eucommia ulmoides, Scleromitrion diffusum, Radix Rehmanniae, Semen Sojae Nigrum, Rhizoma Dioscoreae, American Ginseng, Alisma plantago-aquatica, Rhizoma Smilacis Glabrae, Leonurus japonicus, and Radix Platycodonis. Its main ingredients are Tanshinone IIA, Ginsenoside Rb1, Ginsenoside Re, etc. It has effects on nourishing qi and yin, strengthening spleen and kidney, clearing residual toxins, and is commonly used in patients with chronic nephritis, proteinuria, and hematuria to improve symptoms such as edema, fatigue, dizziness, and tinnitus in clinical practice (60). Specifically, Wang X. et al. (74) used network pharmacology and metabolomics techniques to jointly elucidate the mechanism of SKT in improving DKD. The results showed that SKT can inhibit IR and correct abnormal metabolism in DKD mice through the starch sucrose metabolism pathway and the biosynthesis of unsaturated fatty acids. It mainly inhibits renal inflammation by regulating the TNF signaling pathway and Toll-like receptor signaling pathway, with IL-6, Mitogen-activated protein kinase-3 (MAPK3), vascular endothelial growth factor (VEGF), TNF, etc. being its main targets of action. In addition, Chen Q. et al. (75) found that SKT inhibits the expression of inflammatory mediators nuclear factor-κB (NF-κB), TNF-α, and IL-1β, and its anti-inflammatory activity goes hand in hand with the abundance of gut microbiota.

Glutathione is an effective antioxidant substance within cells, which can improve the functions of the glomeruli and renal tubules. When used in combination with SKT, it can significantly reduce the TC level in patients with DKD and remarkably improve urinary protein (76). Alprostadil is commonly used clinically to improve microcirculation. When combined with SKT, it has a better effect on improving the renal function and blood lipids of patients with early-stage DKD compared with using it alone, and it has good safety (77).

#### 3.1.3 Jinshuibao capsule

According to the pharmacopoeia (60), JSB is made by packaging fermented cordyceps powder (CS-4) into capsules. Its main ingredients are uridine, guanosine, adenosine, ergosterol, etc. With the functions of nourishing the lungs and kidneys, and replenishing essence and qi, JSB is commonly used in clinical practice for diseases such as chronic renal insufficiency hyperlipidemia, chronic bronchitis, cirrhosis and other diseases. JSB have therapeutic effects on stable chronic obstructive pulmonary disease (COPD) by increasing FEV1% pred, FEV1/FVC ratio, FEV1, FVC, and PaO2 levels, while reducing PaCO2 levels (78). At the same time, JSB increases the levels of serum SOD and calcitonin gene-related peptide (CGRP) and reduces C-reactive protein (CRP) and endothelin (ET) -1 to enhance the body’s antioxidant capacity and reduce the severity of micro inflammatory reactions in the body and thus has a positive regulatory effect on renal hemodynamics with significant therapeutic effects (79, 80). What’s more, an *in vitro* experiment (81) has shown that fermented cordyceps has significant renal protective effects, which are achieved by facilitating proliferation and hindering apoptosis of proximal renal tubular cells, possibly through targeting Caspase-3, Bcl-2-associated X protein (Bax), VEGFA, and phosphatase and tensin homolog deleted on chromosome ten (PTEN). The protein kinase B (AKT) and extracellular regulated protein kinases (ERK) signaling pathways might be the key mechanisms for the therapeutic efficacy of fermented cordyceps in treating DKD.

Ramipril belongs to ACEI, which can effectively reduce the intraglomerular pressure and decrease the excretion of urinary protein (82). The combination of JSB and ramipril in the treatment of patients with DKD can significantly reduce urinary protein, regulate the disordered lipid metabolism, and have a positive regulatory effect on renal hemodynamics, with obvious therapeutic effects (80). Moreover, The combination of JSB with telmisartan and candesartan cilexetil can protect renal function, improve blood lipid levels, effectively slow down the progression of diabetic kidney disease (DKD), and its therapeutic effect is significantly better than that of using western medicine alone (83, 84). Sulodexide can achieve the goal of treating early-stage DKD through multiple pathways, such as improving the hypercoagulable state of the body’s blood, renal blood perfusion, reducing proteinuria, and antagonizing the apoptosis of glomerular cells (85). When combined with JSB, it can significantly reduce the urinary albumin excretion in patients with early-stage DKD, protect renal function, correct lipid metabolism disorders, improve endothelial function, alleviate the micro-inflammatory state, enhance the body’s antioxidant capacity, and regulate renal hemodynamics (79).

#### 3.1.4 Huangkui capsule

HKC, extracted from Abelmoschus manihot, can promote diuresis, remove blood stasis, and clear heat. Its main component is flavonoids, which effectively treat damp heat syndrome and DKD (damp heat combined with blood stasis) (86–88). Clinical studies demonstrated that HKC can improve renal function and lipid metabolism disorders in DKD patients significantly. The mechanism is probably reducing serum MDA, increasing serum GSH-Px and SOD, inhibiting oxidative stress, and thus exerting a renal protective effect (89, 90). Moreover, HKC treatment for DKD has a significant anti-inflammatory effect, manifested in the reduction of TNF-α, hs-CRP, IL-2, IL-6, IL-8, IL-1β, γ-glutamyl transferase (GGT), and advanced oxidation protein product (AOPP) levels, thereby increasing capillary permeability, reducing edema, improving inflammatory response and oxidative stress status in the body, ultimately alleviating kidney damage (91–96). Animal experiments have shown that HKC treatment in DKD rats can improve kidney morphology and weight, as well as renal hypertrophy index, and alleviate glomerular hypertrophy, GBM pachynsis, and mild renal interstitial dilation. At the same time, podocyte foot process fusion is reduced, the proliferation of capillary endothelial cells is mild (97, 98). HKC increased the expression of PPARα and its target genes (such as LPL and aP2), PPARγ and its target genes (such as LPL, CPT-1, ACO, and CYP4A) in the liver and kidneys of DKD rats. These genes participate in entry, oxidation and hydroxylation of fatty acids, which may lead to the fall-off in fatty acid exposure in the kidneys of DKD rats (99). Therefore, these influences may trigger a decrease in the synthesis and secretion of triglycerides. In addition, the secreted protein acidic and rich in cysteine (SPARC) is secreted by adipocytes and its expression and secretion are impacted by fat mass, leptin, insulin, and glucose, and it is involved in the pathogenesis of tumorigenesis and renal and liver fibrosis (100, 101). HKC can also inhibit SPARC levels, inhibit Klotho, downregulate phosphorylated p38MAPK and phosphorylated Akt (p-Akt) in DKD rats to alleviate renal fibrosis (102–104). Furthermore, HKC can alleviate epithelial-mesenchymal transition (EMT) in renal tubules of DKD rats by suppressing the activation of NLRP3 inflammasome and TLR4/NF-κB signaling transduction in the kidneys (105). W. Wu et al. (98) found that HKC can restrain the phosphorylation of Akt, mTOR and p70S6K in the kidneys, as well as the overexpression of TGF-β1 protein. Its active ingredient, hyperoside, can inhibit the phosphorylation of PI3K, Akt, mTOR and p70S6K in murine mesenchymal cells induced by high glucose, further demonstrating that HKC can safely and effectively relieve early DKD glomerular pathological changes. Additionally, HKC can readjust the activity of solute carriers (SLC) in both renal proximal and distal tubules, thereby inhibiting the development of DKD (106).

A Meta-analysis (107) has shown that the combination of HKC and ARB/ACEI drugs has obvious advantages in the treatment of early-stage DKD. It significantly improves the clinical treatment efficiency, and is more effective in reducing the levels of urinary albumin excretion rate (UAER), serum creatinine (Scr), blood urea nitrogen (BUN), TG and TC. Moreover, it does not increase the probability of occurrence of adverse reactions. Another systematic review also elaborated the efficacy of HKC combined with Western medicine in the treatment of DKD (108),

#### 3.1.5 Yi-Shen-Hua-Shi granule

YSHS is composed of Panax ginseng, Astragalus membranaceus, Atractylodes macrocephala, Poria cocos, Panax quinquefolius, Pinellia ternata, Hansenia weberbaueriana, Angelica pubescens, Saposhnikovia divaricata, Bupleurum chinense, Coptis chinensis, Paeonia lactiflora, Citri reticulatae pericarpium, Radix glycyrrhizae preparata, Zingiberis rhizoma recens and Jujubae fructus, which is good at promoting yang and tonifying the spleen, nourishing kidneys and removing dampness, promoting diuresis and reducing swelling (109). It is commonly used in clinical practice to treat patients with chronic glomerulonephritis (Scr<2mg/dl) and improve their symptoms such as proteinuria, edema, and cold sensitivity. Its main ingredients are Ginsenoside Rg1, Ginsenoside Re, Ginsenoside Rb1 and Astragaloside (60). There are studies indicating that YSHS have the potential to treat IgA nephropathy and sepsis induced acute kidney injury (110, 111). After treatment, SOD levels increased; MDA, TNF-αand IL-6 levels decreased, and insulin-producing beta cell function index increased, suggesting that its mechanism perhaps has a bearing on improving oxidative stress levels and increasing insulin beta cell function index (112). Shen Zhen et al. (109) found that six pathways were simultaneously enriched through a combined analysis of metabolomics and transcriptomics, consisting of glycerophospholipid metabolism, arachidonic acid metabolism, purine metabolism, primary bile acid biosynthesis, ascorbic acid and aldehyde metabolism, and galactose metabolism. What’s more, studies have indicated that YSHS can improve gut microbiota translocation, regulate gut microbiota structure, increase microbiota diversity and increase the abundance of probiotics such as lactobacilli (which is instrumental in reducing fasting blood glucose, ameliorating glycolipid metabolism, alleviating insulin resistance and reducing tissue damage) (113) through the “gut kidney axis” in DKD rats, thereby improving glucose and lipid metabolism, lowering inflammation levels, lowering urinary toxins in the body, and then protecting kidney function (114, 115). YSHS can regulate the blood sugar and lipid levels of DKD rats, alleviate the symptoms of IR and podocyte reduction, and is conducive to the repair of GFB function, possibly correlated with the increase of nephrin and podocin protein levels and the decrease of platelet-derived growth factor receptorsβ (PDGFRβ) protein levels (116).

Clinical studies have suggested that YSHS can safely and effectively improve clinical symptoms, renal function, and blood lipid metabolism levels in DKD patients. It is more effective than using Western medicine alone, such as calcitriol, ligustrazine hydrochloride, and sulodexide. When combined with Tripterygium wilfordii Hook. f. polysaccharide tablets, YSHS has a synergistic and detoxifying effect (112, 117–123).

#### 3.1.6 Shenmai injection

SMI is made by mixing extracts of talinum paniculatum, radix ophiopogonis, etc. Among them, talinum paniculatum greatly replenishes qi, restores the pulse condition and solidifies the body, and enriches qi and blood; radix ophiopogonis nourishes yin and moistens the lungs, benefits the stomach and generates fluids, clears the heart and eliminates annoyance (124). Compatibility of the two has the functions of nourishing qi, strengthening the body constitution, nourishing yin and generating fluids, and promoting blood circulation. Modern pharmacological studies have demonstrated that the major active ingredients of SMI are Ginsenoside Rb1, Ginsenoside Rg1, Ginsenoside Re, and Saponin D, which interact directly with IL-6/STAT3 directly (125). In clinical practice, SMI is widely used for various diseases, such as ischemic stroke, viral myocarditis, and so on (126, 127).

A randomized controlled trial (128) involving 68 patients with DKD showed that the combination of SMI and alprostadil treatment can significantly improve the relevant indicators of qi deficiency and blood stasis type DKD patients, namely Scr, blood urea nitrogen (BUN), UAER, TC, TG, and effectively improve blood lipids and renal function in DKD patients.

Additionally, Qiyao Xiaoke Capsule (129), Soft Capsules of Compound Oil of Jujube, Arboruitae and Gardenia (130), Liuwei Dihuang Pills (131), Keluoxin Capsules (132), Compound Danshen Dropping Pills (133), Shenqi Jiangtang Granules (134), Tianqi Jiangtang Capsules (135, 136), these traditional Chinese patent medicines have been proved to improve the lipid metabolism disorder in DKD patients or animal models, improve renal function, and delay disease progression.

### 3.2 Traditional Chinese medicine compound combined with Western medicine in the treatment of DKD with lipid metabolism disorder

The traditional Chinese medicine compound is a clinically effective formula composed of two or more single herbs used according to the principles of TCM syndrome differentiation and treatment and the principles of composition and prescription. Specifically, it is formulated according to the “Monarch, Minister, Assistant and Guide” compatibility principles.

#### 3.2.1 Tangshen formula

TSF is composed of Astragalus membranaceus, Ghost arrow feather, Rehmannia glutinosa, Fructus aurantii, Cornus officinalis, Rhubarb, and Panax notoginseng (137). Qin et al. (138) identified representative components of TSF, including loganin, calycosin-7-O-b-D-glucoside, naringenin-7-rhamnosidoglucoside, neohesperidin, naringenin and aloeemodin, by high-performance liquid chromatography (HPLC), which improved the blood lipid levels and reduced liver steatosis in db/db mice. Molecular biology studies (139, 140) have shown that TSF can significantly inhibit urinary albumin excretion and relieve kidney damage in DKD rats. It downregulates the expression of pro-inflammatory factors: GSDMD, NLRP3, IL-1β, IL-18 and Caspase-1 *in vivo*, and blocks renal inflammation driven by NF-κB and renal fibrosis mediated by TGF-β/Smad3 through blocking the Smad7 degradation pathway mediated by Smurf2, preventing apoptosis of renal tubular epithelial cells through regulating the TXNIP-NLRP3-GDMD axis *in vitro*. Liu Ping et al. (141) found that TSF treatment reduced serum LDL-C, TC and TG levels in db/db mice, while HDL-C levels had no significant effect. Upregulation of ABCA1 reduced cholesterol accumulation in the kidneys of db/db mice, indicating that the promotion of cholesterol efflux mediated by ABCA1 may contribute to the therapeutic effect of DKD.

A multi-center, randomized, double-blind, placebo-controlled clinical study has shown that the combination of TSF and irbesartan has demonstrated good effects in reducing proteinuria and increasing the estimated glomerular filtration rate in patients with DKD. Among them, the blood lipid levels of patients with massive proteinuria have been improved, while in patients with microalbuminuria, only the LDL has shown a significant change, and there are no significant differences in other blood lipid levels (142).

#### 3.2.2 Danggui Buxue decoction

DBD is a classic formula for nourishing qi and blood, composed of Astragalus membranaceus and Angelica sinensis Radix in a ratio of 5:1. In this formula, Astragalus membranaceus is used to nourish the lungs and spleen, nourishing the source of biochemistry. Angelicae sinensis Radix is used to supplement blood and nourish the body. Thus, tangible blood is born from intangible qi, and replenishing qi generates blood (143). Moreover, a study on the application of DBD in combination with Jingui Shenqi Pill in 80 DKD patients indicated that the combination of the two could decrease the Scr, urea nitrogen and 24-hour urine protein content more than using Jingui Shenqi Pill alone. At the same time, TC, TG, LDL-C diminished, and HDL-C dramatically increased (144). A molecular study (145) has shown that DBD can alleviate endoplasmic reticulum stress in DKD rat models by inhibiting PERK pathway related proteins, protecting kidney tissue and function, improving TC and TG, and even improving MAU and HDL at high doses compared to gliclazide (146). A network pharmacology study (147) based on lipidomics and transcriptomics has shown that the improvement effect of DBD on DKD is cardinally bound up with adjusting glycerophospholipid and sphingolipid metabolism, attenuating inflammation and IR. However, the active ingredients of DBD may act on VEGFA, ACE, NOS2, NOS3 and MAP3K5 to downregulate the expression of Degs2 and Cers genes, reduce the amount of pc, PEs, Cers, and SMs, thereby alleviating the symptoms of DKD, some of which involve AGE-RAGE, sphingolipids and many kinds of inflammation related signaling pathways.

A study with DBD as the treatment group and irbesartan as the control group shows that DBD can improve the renal function and blood lipid levels of DKD patients with qi deficiency and blood stasis (148). Studies have shown that DBD combined with conventional Western medicine therapy has significant therapeutic effects on patients with DKD. It can effectively reduce blood lipids and proteinuria levels, inhibit systemic inflammatory reactions, improve renal blood circulation, and defend renal function (143).

#### 3.2.3 Tangshenkang

TSK is made of Astragalus membranaceus, Cornus officinalis, Rehmanniae Radix, Common yam rhizome, Poria cocos, Polygonatum sibiricum, Atractylodes lancea, Ligusticum sinense, Prunus mume, Hirudo nipponica whitman and other medicines. It functions in supplementing qi, nourishing yin, invigorating blood circulation and removing blood stasis. As early as 1999, Song Haixiang and others (149, 150) discovered that TSK has an improving effect on renal function indicators and control of blood lipids in DKD patients, and directly inhibits the function and secretion of IL-6 and TGF- β 1 to protect the kidneys. Animal experiments suggested that TSK can significantly improve urinary microalbumin, urinary creatinine, 24-hour urinary protein, and renal function in DKD rats and mice, also improve blood glucose and lipids levels to varying degrees (151–153). Moreover, TSK can significantly alleviate the pathological changes compared to Benazepril in kidneys of DKD rats, inhibit the expansion of mesangial area and glomerular area (154), significantly downregulate TNF-α values, increase SOD levels and reduce MDA levels, which is related to its anti-inflammatory effect and the reduction of oxidative stress levels (151). Shi Xiaowei et al. (152) found that TSK reduced the expression of α-SMA, TGF-β, and CD8+ in the kidneys of DKD mice, decreased ECM deposition in kidney tissue, and alleviated the degree of renal interstitial fibrosis and glomerulosclerosis.

Subsequently, multiple clinical studies have shown that taking TSK in addition to conventional Western medicine treatment can significantly improve renal function indicators, urinary microalbumin excretion, blood lipid levels, reduce blood viscosity, and effectively postpone disease progression of DKD patients (155, 156).

Besides, Huangqi Tang has shown potential in improving lipid metabolism disorders related to DKD by adjusting the expression of lipid metabolism interrelated genes and improving lipid mass spectrometry (157, 158). Xiaoke Shen`an Decoction may affect the activation of subsequent biological pathways by acting on RAF1 and BCL-2 targets, and improve lipid metabolism and renal function in DKD patients, thereby inhibiting the progression of DKD (159, 160). However, further research is needed on the efficacy and safety of the combination with Western Medicine.

### 3.3 Single Chinese medicine

TCM often exerts its efficacy through compatibility, so single TCM preparations are rarely used in clinic. However, owing to the complexity of pharmacology, the exact latent mechanisms of traditional Chinese medicine preparations are difficult to distinguish. In order to avoid adverse reactions, a single Chinese medicine is more suitable for elucidating the exact mechanism of action of DKD.

#### 3.3.1 Astragalus membranaceus

As is the dried root of the leguminous plants Astragalus Membranaceus (Fisch.) Bge. var.mongholicus (Bge.) Hsiao or Astragalus Membranaceus (Fisch.) Bge. The main active ingredient of AS is Astragaloside IV, which has a mild temperature and a sweet taste. It has the effects of tonifying qi and promoting yang, strengthening exterior and reducing sweat, inducing diuresis for removing edema, generating fluids and nourishing blood, breaking stagnation and unblocking rheumatism, supporting toxins and eliminating pus, and promoting healing and muscle growth (60). Among them, AS IV improves DKD by counteracting oxidative stress, restoring mitochondrial homeostasis (inhibiting Drp-1 and PINK1/Parkin signaling pathways), weakening ERS, adjusting calcium homeostasis, relieving inflammation, and improving EMT and endothelial function (161). Proteomic studies have shown that AS IV treatment possibly reduces lipid deposition, lipid related protein and metabolite levels in the kidneys affected by DKD, which perchance defers the occurrence and progression of DKD through adjusting HMOX1 mediated lipid metabolism disorders (162). Part AS II enhances the ability of resistance to oxidative stress through the co - regulation of Nrf2 and PINK1, has beneficial effects on autophagy, and improves podocyte injury and mitochondrial dysfunction in diabetes rats induced by STZ (163).

#### 3.3.2 Panax Notoginseng

According to the pharmacopoeia (60), Panax Notoginseng (Burk.) F.H. Chen, a plant in the Araliaceae family, is derived from the dry root and rhizome with a warm nature, sweet taste, and slight bitterness. It is used for dispersing blood stasis, stopping bleeding, reducing swelling and relieving pain. Its main ingredients are ginsenosides Rg1, Rb1, and R1. Xue Rui et al. (164) found that total saponins of Panax notoginseng can significantly improve proteinuria, mesangial dilation, podocyte apoptosis and morphological changes in DKD rats, and some of them exert antioxidant and anti-apoptotic effects by regulating the PTEN-PDK1-Akt mTOR signaling pathway and reducing the expression of Nox4, while reducing ROS markers such as 8-OHdG and 4-hydroxy-2-nonenal. Furthermore, Shenyilan et al. (165) demonstrated that Panax notoginseng saponins Fc, a novel saponin isolated from PNG, partially improved mitochondrial dysfunction and pyroptosis in GECs by regulating the HMGCS2 pathway. Moreover, researches have shown that Panax notoginseng polysaccharides (PNPs), the main byproduct of Panax notoginseng saponin extraction, have effects such as regulating lipid metabolism disorders, reducing inflammation, enhancing the body’s immunity, anti-tumor, anti-aging, etc (166). Li Yi et al. (167) found that PNPs can significantly improve IR levels and renal function in DKD rats, diminish the expression of inflammatory factors such as IL-1β, IL-6 and TNF-α, and also regulate lipid metabolism disorders through downregulating the levels of SREBP-1c and ACCα, reducing blood fat content, ultimately achieving the effect of treating kidney damage in T2DM rats.

#### 3.3.3 Salvia miltiorrhiza

SM is the dried root and rhizome of Salvia miltiorrhiza Bge, a plant in the family Lamiaceae. Its main components are tanshinones, which are bitter in taste and slightly cold in nature (60). It has the functions of invigorating blood circulation, removing blood stasis, relieving pain, clearing the heart and eliminating annoyance, cooling the blood and eliminating carbuncles (168). Pharmacological studies have indicated that multiple active ingredients in SM can improve lipid metabolism disorders, such as Salvianolic acid A (SalA), which can improve lipid levels in STZ induced SD rats (169). Sal B can reduce total cholesterol and triglyceride levels and regulate dyslipidemia by regulating downstream effectors of AMPK, such as PPARα and acetyl CoA carboxylase (170). Clinical studies have shown that intravenous infusion of salvianolic acid salt can lower TC, TG, LDL-C, and increase HDL-C levels in DKD patients (171). At the same time, it can effectively reduce inflammatory response and alleviate oxidative stress through reducing CRP, TNF-α, IL-6, ROS, MDA levels and increasing T-AOC, SOD, GSH Px levels (172, 173). Tanshinone IIA can inhibit pyroptosis by adjusting the thioredoxin interacting protein Txnip/NLRP3 inflammasome, thereby retarding the progression of DKD. It can also affect the distribution of high-density lipoprotein subfractions in rats, thereby regulating cholesterol metabolism and reducing lipid deposition (174). Z. Xu et al. (175) discovered that the combination of salvianolic acid B and tanshinone IIA has a synergistic anti-inflammatory function, reforming glucose and lipid metabolism abnormalities and liver and kidney damage in early DKD rats. It improves urinary and serum metabolism disorders by unsaturated fatty acid biosynthesis, glycerophospholipid metabolism, steroid biosynthesis, alanine and arachidonic acid biosynthesis, and its mechanism is possibly bound up with the PI3K/Akt/NF-κB signaling pathway. What’s more, sodium tanshinone IIA sulfonate can be obtained by sulfonation of tanshinone IIA, which is currently the only monomeric chemical drug in China prepared from the lipophilic active ingredient of SM. It can significantly improve the blood lipid levels of DKD hemodialysis patients by inhibiting the reaction of NLRP3 to suppress the splicing of caspase-1, thereby reducing the maturation and release of IL-1β, achieving anti-inflammatory, anti-oxidant, and anti-pyroptosis effects, and delaying the progression of DKD (176–178).

Besides, ligustri lucidi fructus (LLF) may affect the progression of DKD through inflammatory response (179). Moreover, the steamed LLF extract exhibits a stronger protective effect on the kidneys. This is manifested in the improvement of indicators such as Scr, BUN, and 24-hour urinary protein. It also shows better control of hyperlipidemia and a reduction in the release of pro-inflammatory mediators like TNF-α, IL-6, and IL-1β in DKD rats (180). Hirudin, the main active ingredient of Hirudo nipponica Whitman (HNW), is a natural thrombin inhibitor that can improve kidney injury and inhibit inflammation through the p38/NF-κB pathway in the DKD rat model (181). HNW freeze-dried powder has a dose-dependent effect on improving renal function in DKD rats, while TC, TG, and LDL are all reduced, with no statistically significant difference in HDL (182).

## 4 Discussion

DKD, as a common microvascular complication of diabetes, has become the main cause of ESRD. Its pathogenesis is complex, with multiple etiologies interacting with each other. Among them, lipid metabolism disorder is an important risk factor for the occurrence and development of DKD. Therefore, it is recommended to intervene in lifestyle and adjust medication while receiving routine medication treatment, and control blood lipid levels, thus effectively retarding the progression of renal disease. Along with the modernization of TCM, many kinds of traditional Chinese patent medicines, compound Chinese medicines and single Chinese medicine preparations are constantly gaining increasing attention, and their therapeutic effects and safety have also been largely verified (**Table 1**). Currently, there is no specific medicine for the clinical therapy of DKD, and Western medicine has a single mode of action and many adverse reactions. Many drugs cannot be used for a long time. Numerous researches have suggested that the combined use of TCM and Western medicine often yields better results than using TCM or Western medicine alone (**Table 2**). In general, it is of great significance to further pinpoint the pathogenesis of DKD combined with lipid metabolism disorders and better apply integrated Traditional Chinese and Western medicine to treat DKD.

**Table 1:** The summary of TCM.

Type	Name	Main ingredients	Indication	Mechanism of action
Traditional Chinese patent medicine	Bailing Capsule	mannitol, adenosine, total amino acids (60)	Chronic renal insufficiency (60)	Anti-inflammatory Antioxidant Inhibit adipogenesis and enhance lipolysis (63, 64)
	Shenyan Kangfu Tablets	Tanshinone IIA, Ginsenoside Rb_1_, Ginsenoside Re (60)	Chronic nephritis Proteinuria Hematuria (60)	Improved IR Anti-inflammatory (74, 75)
	Jinshuibao Capsule	uridine, guanosine, adenosine, ergosterol (60)	Chronic renal insufficiency Hyperlipidemia (60)	Antioxidant Anti-inflammatory Promote proliferation and anti-apoptosis (79–81)
	Huangkui Capsule	flavonoids (86, 87)	Damp heat syndrome and DKD (damp heat combined with blood stasis) (88)	Anti-inflammatory Antioxidant Anti-fibrosis Alleviate EMT (89–96, 102–105)
	Yi-Shen-Hua-Shi granule	Ginsenoside Rg_1_, Ginsenoside Re, Ginsenoside Rb_1_ and Astragaloside (60)	chronic glomerulonephritis Proteinuria (60)	Improve IR Anti-inflammatory Antioxidant (112, 113, 116)
	Shenmai injection	Ginsenoside Rb_1_, Ginsenoside Rg_1_, Ginsenoside Re, and Saponin D (125)	Shock and coronary disease Chronic pulmonary cardiac disease (125)	Anti-inflammatory (125)
Traditional Chinese Medicine Compound	Tangshen Formula	Loganin, calycosin-7-O-b-D-glucoside, naringenine-7-rhamnosidoglucoside, neohesperidin, naringenin, aloeemodin (138)	DKD with proteinuria (140)	Anti-inflammatory Anti-fibrosis (139, 140)
	Danggui Buxue Decoction	Ferulic acid, Caffeic acid, Ligustilide, Calycosin, Formononetin, Butylphthalide, Astragaloside IV (147)	Disease of Qi and blood deficiency (147)	Anti-inflammatory Ameliorate ER stress Improve IR (145, 147)
	Tangshenkang		DKD (151)	Anti-inflammatory Antioxidant Anti-fibrosis (151)
Single Chinese Medicine	Astragalus membranaceus	Astragaloside IV (60)	Deficiency of qi in DKD Endogenous heat and wasting-thirst (60)	Antioxidant Anti-inflammatory Anti-fibrosis Alleviate EMT Regulate mitochondrial homeostasis (161–163)
	Panax notoginseng	Ginsenosides Rg_1_, Ginsenosides Rb_1_, Ginsenosides R_1_ (60)	Blood stasis in DKD (60)	Antioxidant Anti-inflammatory Anti-apoptosis Improve mitochondrial dysfunction (164–167)
	Salvia miltiorrhiza	tanshinones (60)	Blood stasis and abdominal pain in DKD (60)	Antioxidant Anti-inflammatory Anti-apoptosis (176–178)

**Table 2:** Clinical research.

Name of TCM	Author	Number	Group	Time	Outcome
Bailing Capsule (BC)	He et al., 2023 (69)	76	Control: Irbesartan Trial: Irbesartan+BC	8w	①②⑤⑥⑭⑮⑯
	Huang et al., 2016 (68)	118	Control: Losartan Trial: Losartan+BC	3m	①②③④⑦⑩⑭⑮⑯⑰
	Jin 2016 (67)	100	Control: Valsartan Trial: Valsartan+BC	12w	①②⑤⑥⑩⑫⑭
	Fang et al., 2022 (70)	80	Control: Dapagliflozin Trial: Dapagliflozin+BC	8w	①②③④⑤⑥⑧⑩⑰
	Niu 2020 (71)	170	Control: Pancreatic kininogenase Trial: Pancreatic kininogenase +BC	8w	①②③④⑤⑧⑰⑱
	Hu et al., 2018 (72)	210	Control: Simvastatin Trial: Simvastatin+BC	3m	①②③⑤⑥⑧⑰
	Zhong et al., 2020 (73)	112	Control: Atorvastatin Trial: Atorvastatin+BC	6m	①②③④⑤⑥⑦⑰㉔㉕
	Gao et al., 2020 (64)	102	Control: Sulodexide Trial: Sulodexide+BC	8w	①②⑭㉑㉒㉓
	Tan 2021 (63)	78	Control A: Healthy adults Control B: Regular treatment Trial: Regular treatment + BC	12w	①②③④⑤⑥⑦⑩⑭⑰㉔㉕
Shenyan Kangfu Tablets (SKT)	Zhang et al., 2016 (76)	100	Control: Reduced glutathione Trial: Reduced glutathione+SKT	15d	①②⑤⑥⑧⑩⑬⑭⑮⑲
	Cheng et al., 2018 (77)	120	Control: Alprostadil Trial: Alprostadil+SKT	8w	①②⑤⑥⑧⑲
Jinshuibao Capsule (JSB)	Jiang2021 (80)	138	Control: Ramipril Trial: Ramipril+JSB	2m	②③⑤⑥⑦⑧⑰㉓㉑
	Pan et.al2016 (83)	80	Control: Telmisartan Trial: Telmisartan+JSB	3m	①②③④⑤⑥⑩⑪⑫⑲
	Wang et.al2020 (84)	78	Control: Candesartan Trial: Candesartan+JSB	8w	①②③④⑤⑥⑩⑫⑯⑱⑲
	Li et.al2019 (79)	80	Control: sulodexide Trial: sulodexide+JSB	4m	②③⑤⑥⑦⑧⑰㉓㉑
Huangkui Capsule (HKC)	Cao et al., 2011 (89)	106	Control: Regular treatment Trial: Regular treatment+HKC	24w	①②③
	He et.al2024 (90)	80	Control: Regular treatment Trial: Regular treatment+HKC	6m	②③⑦⑪⑭⑮⑯㉒㉓
	Li et.al2014 (91)	84	Control: Regular treatment Trial: Regular treatment+HKC	8w	①②③④⑤⑥⑭⑮⑯⑰㉔㉕
	Feng et.al2016 (92)	120	Control: Regular treatment Trial: Regular treatment+HKC	8w	②⑤⑥⑧⑭⑰⑲㉔㉕
	Sun at.al2018 (93)	128	Control: Reduced glutathione Trial: Reduced glutathione+HKC	8w	①②③⑤⑦⑧⑫⑭⑮⑯㉕
	Wei 2020 (94)	92	Control: Captopril+Compound α-Ketoacid Tablets Trial: Captopril+Compound α-Ketoacid Tablets +HKC	2m	①②⑤⑥⑭㉔㉕
	Zhao et.al2021 (95)	150	Control: Valsartan Trial: Valsartan+HKC	2m	①②③④⑤⑥⑦⑰㉔
	Xu et.al2021 (96)	116	Control: Simvastatin Trial: Simvastatin+HKC		①②⑤⑥⑦⑭⑯㉕㉔㉖
Yi-Shen-Hua-Shi granule (TSHS)	Li et al., 2013 (117)	30	Control: Irbesartan Trial: Irbesartan+YSHS	4m	①②⑤⑧⑭⑮⑲⑳
	Zhang et.al2014 (118)	120	Control: Sulodexide Trial: Sulodexide+YSHS	4m	①②③④⑤⑥⑦⑭⑰㉕
	Cheng et.al (119)	98	Control: Alprostadil Trial: Alprostadil+YSHS	2m	①③④⑤⑥⑫⑭⑮⑯⑱
	Hu2018 (120)	134	Control: Sulodexide Trial: Sulodexide+YSHS	4m	①②③④⑤⑦⑭⑮㉑㉒㉓
	Chen2018 (121)	140	Control: Sulodexide Trial: Sulodexide+YSHS	12w	①②③④⑤⑥⑦⑧㉑㉒㉓
	Fu et.al2020 (122)	92	Control: Tripterygium wilfordii polyglycosides Tablet Trial: Tripterygium wilfordii polyglycosides Tablet+YSHS	3m	①②⑤⑥⑩
	Fu et.al2021 (123)	150	Control: Ligustrazine Hydrochloride Trial: Ligustrazine Hydrochloride+YSHS	12w	①②③④⑤⑥⑩⑭⑰㉔㉕
	Liu et.al2023 (112)	120	Control: Calcitriol Trial: Calcitriol+YSHS	2m	①③④⑤⑥⑦⑫⑭⑱㉒㉓㉔㉕
Shenmai injection (SMI)	Chen2016 (128)	68	Control: Alprostadil Trial: Alprostadil+SMI	14d	①②⑤⑥⑦
Qiyao Xiaoke Capsule	Wang et al., 2013 (129)	79	Control: Regular treatment Trial: Regular treatment+QXC	12w	①②④⑩⑭
Compound Danshen Dropping Pills	Lvu et.al2023 (133)	72	Control: Irbesartan Trial: Irbesartan+CDDP	4m	①②③④⑤⑥⑦⑧
Shenqi Jiangtang Granules	Wu2016 (134)	46	Control: Irbesartan Trial: Irbesartan+SJG	10w	①②③④⑤⑥⑦㉕㉖㉗
Tianqi Jiangtang Capsules	Tang et.al2016 (135)	108	Control: Regular treatment Trial: Regular treatment+TJC	8w	①②③④⑤⑥⑭⑮⑯
	Hou2017 (136)	98	Control: Regular treatment Trial: Regular treatment+TJC	8w	①②③④⑤⑥⑭⑮⑯
Tangshen Formula (TSF)	Li et al., 2015 (142)	180	Control: Placebo Trial: Placebo+TSF	24w	①②③④⑤⑥⑦⑧⑨
Danggui Buxue Decoction (DBD)	Zhong et.al2022 (148)	60	Control: Irbesartan Trial: Irbesartan+DBD	6m	①②⑤⑥⑧⑭⑯
	Li et.al2020 (144)	80	Control: Jingui Shenqi Pill Trial: Jingui Shenqi Pill+DBD	1m	①②③④⑤⑥⑧㉑
Tangshenkang (TSK)	Song et.al1999 (149)	90	Control: Captopril Trial: Captopril+TSK	3m	①②④⑤⑦⑧⑫⑭⑯㉕
	Song et.al2004 (150)	148	Control: Captopril Trial: Captopril+TSK	6m	①②③④⑤⑥⑧⑫⑬⑭⑯㉖
	Zhang et.al2016 (155)	90	Control: Regular treatment Trial: Regular treatment+TSK	6m	①②③⑤⑯⑲㉑
	Pei et.al2021 (156)	106	Control: Regular treatment Trial: Regular treatment+TSK	8w	①②③④⑤⑥⑦⑫⑭⑮⑯

①Total cholesterol, TC; ②Triglyceride, TG; ③Low-density lipoprotein cholesterol, LDL-C; ④High-density lipoprotein cholesterol, HDL-C;⑤Serum creatinine, Scr; ⑥Blood urea nitrogen, BUN; ⑦Urinary albumin excretion rates, UAER; ⑧24-hour urinary protein, 24hUP; ⑨Estimated glomerular filtration rate, eGFR; ⑩Micro-albunminuria, MALB; ⑪Albumin/creatinine ratio, ACR; ⑫β2-microglobulin, β2-MG; ⑬N-acetyl-β-D-glucosaminidase, NAG; ⑭Fasting blood glucose/Fasting plasma glucose, FBG/FPG; ⑮2-hours plasma glucose, 2hPG; ⑯Glycosylated Hemoglobin-Type A1C, HbA1c; ⑰C-reactive protein/Hypersensitive C-reactive protein, CRP/hs-CRP; ⑱Cystatin C, Cys-c;, ⑲Blood pressure, BP; ⑳K^+^; ㉑Endothelin-1, ET-1; ㉒Malondialdehyde, MDA; ㉓Superoxide dismutase, SOD; ㉔Tumor necrosis factor-α, TNF-α; ㉕Interleukin-1β/2/6/8/16, IL-1β/2/6/8/16; ㉖Transforming growth factor-β, TGF-β1; ㉗Vascular endothelial growth factor, VEGF.
